# Impact of Glycosylation on Effector Functions of Therapeutic IgG †

**DOI:** 10.3390/ph3010146

**Published:** 2010-01-12

**Authors:** Riad Abès, Jean-Luc Teillaud

**Affiliations:** 1 INSERM UMRS 872, Paris, F-75006 France; E-Mail: riad.abes@crc.jussieu.com (R.A.); 2 Cordeliers Research Center, Université Pierre & Marie Curie, UMRS 872, Paris, F-75006, France; 3 Université Paris-Descartes, UMRS 872, Paris, F-75006 France; 4 Laboratoire français du Fractionnement et des Biotechnologies (LFB), Les Ulis, France

**Keywords:** antibody, Fc receptor, glycosylation, IgG

## Abstract

Human IgG has only one conserved glycosylation site located in the Cγ2 domain of the Fc region that accounts for the presence of two sugar moieties per IgG. These IgG sugar cores play a critical role in a number of IgG effector functions. In the present review, we describe the main characteristics of IgG Fc glycosylation and some abnormalities of serum IgG glycosylation. We also discuss how glycosylation impacts on monoclonal antibodies (mAbs) and IVIg effector functions and how these molecules can be engineered. Several therapeutic antibodies have now been engineered to be no- or low-fucose antibodies and are currently tested in clinical trials. They exhibit an increased binding to activating FcγRIIIA and trigger a strong antibody-dependent cell cytotoxicity (ADCC) as compared to their highly-fucosylated counterparts. They represent a new generation of therapeutic antibodies that are likely to show a better clinical efficacy in patients, notably in cancer patients where cytotoxic antibodies are needed.

## 1. Introduction

Human IgG has only one conserved glycosylation site located in the Cγ2 domain [asparagine 297 (Asn297)]. The sugar core anchored to this glycosylation site plays a critical role in IgG effector functions and, hence, has been and is still extensively studied [1,2]. Fc-mediated effector functions of IgG include complement activation (leading to complement-dependent cytotoxicity, [CDC]) and engagement of receptors for the Fc region of IgG (FcγRs). Activating FcγR (FcγRI, FcγRIIA, FcγRIIIA) induce antibody-dependent cell cytotoxicity (ADCC), endocytosis of immune complexes followed by antigen presentation, and antibody-mediated phagocytosis. Inhibitory FcγR (FcγRIIB) regulate immune responses by inhibiting the activation of B lymphocytes, monocytes, mast cells and basophils, induced through activating receptors [3,4].

Analyses by X-ray crystallography of human IgG have demonstrated that the carbohydrate chains do not extend into solvent but form a bridge between the two opposing Cγ2 domains [5]. The human IgG hinge region does not contain O-linked glycans as opposed to rabbit IgG, human IgA1 and IgD. Of note, N-linked glycosylation can also be found in variable (V) domains of both heavy (VH) and light (VL) chains of serum IgG and of some monoclonal antibodies (mAbs). This glycosylation pattern has to be taken into account when studying the impact of glycosylation on the effector functions of IgG therapies. Here, we describe the major features of IgG Fc glycosylation, and give an overview of the main serum IgG glycosylation abnormalities. How glycosylation impacts on mAbs and IVIg functions and how these molecules can be optimized by molecular engineering of the sugar moieties are also discussed.

## 2. IgG Fc Glycosylation and Its Impact on IgG/FcγR Interactions

**Figure 1:**
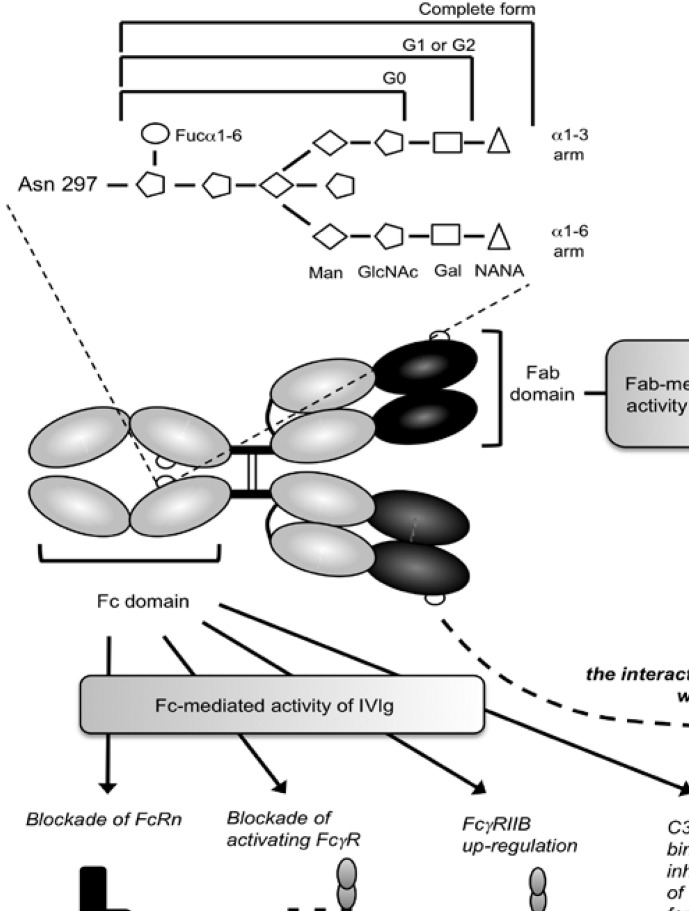
Mechanisms of action of IVIg. Enlarged representation of the Asn297-linked oligosaccharide complex is shown [Fucα1-6: fucose(α1-6); Man: mannose; GlcNAc: N-acetyl-glucosamine; Gal: galactose; NANA: sialic acid]. IVIg mechanisms of action can be divided in two categories: (A) Fab-mediated activity against immunoregulatory or pathogen-related molecules, or presence of anti-idiotype (Id) antibodies that can neutralize autoantibodies and inhibit Id^+^ FcγRIIb^+^ pathogenic B cells. (B) Fc-mediated activity of IVIg through different mechanisms: (i) competitive blockade of FcRn, (ii) competitive blockade of activating FcγR and up-regulation of FcγRIIB and (iii) C3b and C4b binding leading to an indirect inhibition of membrane attack complex (MAC) formation. These molecular mechanisms trigger (i) an increased clearance of pathogenic endogenous antibodies, (ii) the modulation of DC, granulocyte, T and NK cell activity and (iii) a decrease of complement-dependent tissue destruction, respectively. Altogether, Fc-mediated mechanisms ultimately lead to anti-inflammatory activity.

The structure of the carbohydrate chain of the Fc region has been extensively studied in serum IgG, myeloma IgG proteins and mAbs (Figure 1). The chain contains several N-Acetyl-Glucosamine (GlcNAc) and mannose (Man) residues, and eventually galactose (Gal) and fucose (Fuc) residues as well as sialic acid (Sia or NANA for N-acetylneuraminic acid). A GlcNAc, to which a Fucα1-6 is linked or not, is attached to the Asn297. A GlcNAcβ1-4 is attached to this first GlcNAc. A manβ1-4 is then found, to which two Manα1-6 and Manα1-3 arms are attached. Both arms contain an additional GlcNAcβ1-2 to which a Galβ1-4 can be attached or not. Thus, the carbohydrate chain can contain 0, 1 or 2 galactose residues, defining G0, G1, and G2 glycoforms, respectively. Further variations occur, including the presence of a bisecting GlcNAcβ1-4 and the capping of one or both of the terminal galactose residues with a sialic acid or even a Galα1-3 residue.

When studying and manipulating human IgG glycosylation, one has to keep in mind that N-linked glycosylation is also observed in both VH and VL domains of serum IgG (up to 30% in normal serum) and of some mAbs. The glycosylation of V domain is occurring randomly, when hypermutation results in the generation of an Asn/X/Ser(Thr) glycosylation motif (X not being a proline). These glycosylated variable domains are used mostly for anti-carbohydrate antibodies (anti-dextran, anti-levan, anti-Lewis X). This glycosylation could be asymmetrical (only one of the two VH domains being glycosylated), due to a post-translational modification such as a transamidation reaction taking place by transfer of the amide group from the asparagine to a neighbouring glutamic acid. The consequence of asymmetric Fab glycosylation is to generate monovalency and/or non-precipitating antibodies [6]. Fab-associated glycans are extensively sialylated [both mono- (A1) and disialylated (A2)] [7]. The type of the sialic acid linkage may vary depending on the species. Although an α2-6 linkage is observed in human IgG, CHO cells that are commonly used for mAb production only add sialic acid in α2-3 linkage [8]. By contrast, the Fc-associated carbohydrate chains of serum IgG contain low levels of sialic acid (10–15%), whereas therapeutic human IgG1 mAbs exhibit only marginal levels, if any. In addition, mostly monosialylated structures (A1) are present, on the manα1-3 arm. Glycosylation of variable domains can impact positively [9,10] or negatively [10] the affinity of some antibodies. Interestingly, one study reported that the presence of glycans in the VH domain of an anti-factor VIII mAb does not modify its affinity but impacts on its neutralizing capacity in vitro [11]. In this report, the authors suggested that modification of glycosylation in variable domains may provide a novel strategy to modulate the functional activity of therapeutic antibodies.

The most variable residue of the Fc sugar core is the galactose. Its amount is variable depending on the age. In childhood and in elderly people, IgG is less galactosylated. The percentage of G0 glycoforms (agalactosyl IgG) increases in donors older than 50. About 75% of them exhibit between 30 to 50% G0 forms (as compared to 15–30 % in donors < 50). The loss of galactose is at the expense of the G2 forms. It should be emphasized that a two-fold change in % G0 translates into a four-fold change in the % of serum agalactosyl IgG. Due to the inner position of the two carbohydrate chains between the two CH2 domains, the accessibility to the sugar chains is restricted. However, although molecules such as ConA or mannose binding protein cannot bind to native intact IgG, it has been shown that β-galactosidase from *Streptococcus* is able to cleave the galactose residues without heat-denaturation of IgG [12]. Interestingly, the degree of galactosylation does not correlate with the amount of galactosyltransferase in a variety of cell lines producing anti-D antibodies [13], suggesting that other parameters play a critical role in the galactose content of IgG. Hypergalactosylation arises when cells are grown in stationary culture [14] or in low-density static culture [13] as compared to cells grown at high-density in hollow-fiber bioreactor. It has been proposed that the extent of glycosylation depends on the timing of disulphide bond formation [15]. In absence of interchain disulfide bond formation (H-H), the carbohydrate chains could be easily accessible to the glycosyltransferases. On the opposite, the formation of a H-H disulfide bond would limit accessibility of these enzymes, the sugar chains being buried between the two CH2 domains. Thus, the final secreted product in a given cell line would depend on the exact moment and location when/where disulfide bond formation takes place, relative to the glycosylation process, in particular galactosylation. X-ray crystal data have suggested that at least 50% of the Fc-associated chains must be devoid of galactose on the manα1-3 arm to allow the formation of a carbohydrate bridge [5]. However, the examination of a number of monoclonal antibodies has shown that hypergalactosylation (70–80% G2 forms) does occur when cells are grown in static culture [13]. Thus, it appears that there is no strict “pairing” rule with regard to the galactosylation status of IgG. A difference in galactosylation levels has been found when the different human IgG subclasses were analyzed. IgG1 and IgG3 have low G0 levels, while both IgG2 and IgG4 have approximately two-fold higher % G0 levels. 

The impact of glycosylation on IgG effector functions has been examined in numerous *in vitro* experiments using mAbs [16]. In the early 80’s, it was established that deglycosylated IgG no longer bind significantly to FcγRs and to C1q [17], thus being unable to trigger ADCC and complement activation. Monosialylation (A1 form) of IgG1 has been reported to strongly impact on the ability of anti-D mAbs to lyse red cells in ADCC assay [13], while blockade of the processing of the oligomannose intermediate through the terminal glycosylation steps generates IgG1 unable of CDC and exhibiting a four- to six-fold decrease of their Kd for FcγR [18]. In addition, the presence of oligomannose structures has been related to a rapid clearance of IgG from serum, suggesting that these structures are exposed and bind to the mannose receptor expressed by macrophages and other phagocytic cells. Hypergalactosylation positively impacts ADCC mediated by FcγRIIIA (CD16), but does dot modify the ability of IgG to form rosettes with cells expressing the high-affinity activating FcγRI [13]. By contrast, hypogalactosylation leads to poorly active IgG in ADCC assays. It has also been shown that G0 glycoforms have reduced C1q and FcγR binding. A small decrease in the % of galactosylation leads to considerably lower FcγRIIII (CD16)-mediated lysis of red cells [13]. 

## 3. IgG Fc Glycosylation and Immunological Diseases

Low-galactose-containing IgG glycoforms are observed in a number of diseases. It is well established that this decreased galactosylation is restricted to the Fc N-linked oligosaccharides. The % of G0 glycoform (“agalactosyl” IgG) is notably increased in patients with various chronic inflammatory and infectious diseases [rheumatoid arthritis (RA), juvenile chronic arthritis (JCA), active Crohn’s disease, tuberculosis, Lyme disease, sarcoidosis). Since the pioneering work by Mullinax in 1975 showing a decreased galactose content in serum IgG of RA and Systemic Lupus Erythematosis (SLE) patients who also had Sjögren’s syndrome [19], the significance of an elevated presence of G0 glycoforms is still unclear. The most prevalent of the increased G0 IgG diseases is RA. Interestingly, it has been shown that a sudden decrease in the % of the G0 glycoforms parallels pregnancy-induced remission of RA [20]. In addition, the post-partum resumption of the disease correlates with a rapid increase of these forms. Whether these G0 IgG glycoforms, which are likely to exhibit a weak ADCC potency *in vivo*, play a role in the onset of RA or during the development of the disease is still unknown. It should be noted that all IgG subclasses exhibit elevated levels of G0 glycoforms in RA patients. The absence of galactose leaves GlcNAc as the terminal residue, which has allowed the development of assays for agalactosyl IgG. Notably, a mAb (GN7) specifically directed against GlcNAc residues has been used to set up an assay to have a rapid estimation of the amount of agalactosyl IgG [21]. The deficiency in IgG galactosylation in RA patients is likely arising during IgG synthesis and is not due to galactose removal from the blood. It has been suggested that an altered balance of cytokines in these patients could be responsible for the high % of G0 glycoforms and that IL-6 could be directly involved, as its serum level is increased in RA patients and it is a cytokine that acts on B-cell growth. Moreover, by contrast to the effect of a high % G0 glycoform content on FcγRIII-mediated ADCC, it has been shown that removal of galactose has little impact on the binding to both activating FcγRIIA and inhibitory FcγRIIB [13].

Finally, the presence of sialic acid has been related to the properties of an IgM cryoglobulin [22] but another study on an IgG mAb has indicated that its cryoglobulin properties were due to the sialylation of a N-linked oligosaccharide in its Fab portion [23]. 

## 4. Engineering Fc Glycosylation for Optimizing mAb Efficacy

Engineering the Fc region of IgG1 therapeutic mAbs to modulate IgG/FcγRIIIA interactions has become a major goal over the last decade following a number of reports showing that FcγRIIIA play an important role in the efficacy of therapeutic mAbs [24]. Cartron *etal*. [25] showed that FcγRIIIA polymorphism has a profound impact on the response to the treatment with the anti-CD20 mAb rituximab. This important observation was in agreement with the previous work of Clynes *et al*. [26] that demonstrated that therapeutic mAbs require the presence of activating FcγR to control tumor progression and to increase survival rates in mouse models. Thus, since glycosylation is an important parameter for the functions of human IgG [16], strategies to modify the glycosylation profile of human IgG1 or to select the most efficient glycoforms have been extensively explored. This has been achieved by using different techniques, ranging from selection of cell lines that produce IgG with particular glycan composition [27], transfection/transduction of genes encoding well-defined glycosyltransferases [2,28] or disruption of genes encoding other transferases such as FUT-8 in cells like CHO [29]. The fine-tuning of the composition of the culture medium used to grow the transfected cells as well as the use of different culture conditions have been also used to produce recombinant antibodies with better-defined oligosaccharides chains. Of note, despite the evident role played by galactose content in IgG biological activities, most engineering approaches have focused on other residues of the sugar core, in particular on fucose.

Umana *et al*. [30] showed that a human IgG1 mAb with a bisecting GlcNAc induces a strong ADCC as compared to its parental counterpart. It has been then demonstrated that a lack of fucose or a low fucose content in human IgG1 N-linked oligosaccharides markedly increases FcγRIIIA binding and ADCC [27,31,32]. In fact, this latter observation explains why a bisecting GlcNAc induces a strong ADCC,**as the presence of bisecting GlcNAc is always associated with low fucose content. Moreover, the density of the target molecule necessary to the induction of an efficient ADCC is much lower when the IgG1 mAb has a low content in fucose [33,34]. By contrast to FcγRIIIA, no difference of binding to the high-affinity FcγRI (CD64) between these low- and high-fucosylated antibodies is observed. It has been reported also that antibodies without fucose show only a slight increase in their binding to soluble FcγRIIB (sFcγRIIB) ectodomain (ECD) (CD32B) and to the arginine 131-sFcγRIIA ECD polymorphic form (CD32A) [31], if any, when tested by Surface Plasmon Resonance (SPR) assays and/or by ELISA. However, using specific competitive cell binding assays, we have observed that a low-fucosylated anti-D mAb exhibits an increased binding to both membrane activating FcγRIIIA and inhibitory FcγRIIB, as compared to its high fucose counterpart [27]. 

Thus, all these data suggest that changes in the glycosylation profile of IgG or the purification of certain glycoforms of human IgG1 will make it possible to prepare antibodies capable of exerting a fine tuning between the activating and the regulatory functions of FcγR on immune responses. Several therapeutic anti-CD20 antibodies have been engineered to be no- or low-fucose antibodies and are currently tested in clinical trials (Table 1). They exhibit an increased binding to activating FcγRIIIA and trigger a strong ADCC as compared to their highly-fucosylated counterparts. They represent a new generation of anti-CD20 therapeutic antibodies that are likely to show a better clinical efficacy in patients, notably in cancer patients where cytotoxic antibodies are much needed. However, they have to be tested cautiously, as one cannot rule out the emergence of adverse events due to their increased ability to bind activating FcγRIII (CD16). 

**Table 1:** Second generation glyco-engineered anti-CD20 antibodies currently in clinical development.

	GA101	LFB-R603	BLX-301
**Company**	Roche [Glycart]	LFB Biotechnologies [GTC Biotherapeutics]	Biolex
**Format**	Humanized	Chimæric	Humanized
**Type of anti-CD20**	Type II	Type I	NA
**Glyco-engineering**	Low fucose	Low fucose	No fucose / G0
CDC*	↘	=	↘
ADCC**	↗	↗	↗
**PCD°**	↗	=	NA^°°^
**Phase Development**	Phase II	Phase I/II	Pre-clinical
**Indication**	NHL / CLL^+^	CLL	NHL
**Reference**	[51]	[34]	NA

^* ^CDC: complement-dependent cytotoxicity, ^**^ ADCC: antibody-dependent cell cytotoxicity; ^°^ PCD: programmed cell death induction, ^°°^ NA: not available; ^+^ CLL: chronic lymphocytic leukemia.

## 5. Role of Glycosylation in the Inflammatory Activity of IVIg

Intravenous immunoglobulins (IVIg), that are prepared from large pools of plasma originating from thousands of healthy donors, are increasingly being used in the clinic for the therapy of both autoimmune and systemic inflammatory diseases. IVIg are known to interact with numerous components of the immune system, including FcγR, complement, cytokines, lymphocytes, granulocytes, dendritic cells (DCs) or NK cells [35] (Figure 1).

Since IVIg contain a very large repertoire of variable regions present in the normal serum [36], they were first indicated for the treatment of patients with antibody deficiencies. However, aside from the Fab-mediated mechanisms of action of IVIg that might also involve the idiotypic/anti-idiotypic network, IVIg therapeutic efficacy has been also related to the interaction of their Fc portion with FcγR-bearing host immune cells [35,37] (Figure 1). Notably, a human clinical trial that used Fc fragments instead of intact whole IgG to treat children with acute immune thrombocytopenic purpura (ITP) showed that Fc fragments are as efficient as IVIg to increase rapidly platelet levels and to achieve complete or partial clinical responses [38]. Various mouse models have also indicated that the anti-inflammatory/anti-autoimmunity activity of IVIg requires the Fc domain of IgG [39,40,41,42,43]. This Fc-mediated effect may be due to a “competition” effect with endogenous pathogenic antibodies for the binding to FcγR [38] and/or to neonatal Fc receptor (FcRn) [44]. Alternatively, it may be due to the triggering of secondary cellular events, such as FcγR-induced apoptosis or anergy, involving the phosphorylation of immunoreceptor tyrosine-based inhibition motif (ITIM) and immunoreceptor tyrosine-based activation motif (ITAM) [45,46].

To achieve clinical efficacy, IVIg have to be used at high doses. Thus, it addresses the question of whether only a fraction of IVIg preparation is involved in the clinical responses that are observed. IVIg preparation contains tens of IgG glycoforms that might differentially contribute to the therapeutic effect of the preparation. Studies deciphering the specific contribution of some IgG glycoforms have brought some new insights on mechanisms of action of IVIg. It has been shown that sialic acid enrichment of IVIg preparation leads to more than a 10-fold increase of their anti-inflammatory activity [41]. By contrast, removal of sialic acid from the preparation abolishes its therapeutic efficacy. Similarly, sialic acid enrichment of cytotoxic IgG diminishes their efficacy *in vivo,* mirroring their lower affinity for FcγR, and suggesting that most of the anti-inflammatory activity of IVIg is driven by IgG Fc domain. Moreover, a number of studies have demonstrated that the increased therapeutic efficacy of sialic acid-enriched IVIg is dependent on FcγRIIB and possibly, to a lower extent, on FcγRIII [39,40,41,42,43,46]. 

The weak binding activity of monomeric IgG to most of the membrane FcγR led to the hypothesis that IVIg might bind simultaneously both FcγR and some unknown sialic acid-sensitive cell-surface receptor to exert their therapeutic effect [47]. A study hypothesized that colony-stimulating factor 1 [CSF1]-dependent macrophages can specifically bind sialic-acid-rich IgG glycoforms present in IVIg preparation in the spleen [40]. It was therefore postulated that the interaction of sialic-acid-rich IgG glycoforms of IVIg preparation with this unknown sialic-acid sensor receptor might then lead to a trans-upregulation of FcγRIIB on CSF1-dependent macrophages, thus raising the threshold needed for cell activation and the down-modulation of pro-inflammatory processes [48]. A candidate molecule has been identified, a type-C specific lectin expressed by mouse macrophages from the spleen marginal zone, specific ICAM-3 grabbing non integrin-related 1 (SIGN-R1). It is required for the triggering of anti-inflammatory response by sialic acid enriched-Fc portions, including FcγRIIB expression modulation [49]. However, a more recent work reported that the human counterpart of mouse SIGN-R1, namely DC-specific ICAM-3 grabbing non integrin (DC-SIGN), a receptor expressed on DCs, is dispensable for the anti-inflammatory activity of IVIg [50]. Altogether, these data brought new insights on IVIg mechanisms of action and pave the way to new therapeutic approaches using glyco-engineered IVIg or recombinant Fc fragments. These approaches may lead to a decrease in both the dose and the number of injections, overcoming the shortage of therapeutic IVIg in the clinic as recently discussed by Galeotti *et al*. [35].

## 6. Conclusion

It has become increasingly evident that the carbohydrate composition of the glycan linked to asparagine 297 has a profound impact on the binding ability of IgG1 to FcγR and C1q as well as on their functional activity, although it is still unclear how this effect takes place at a molecular level. The enormous efforts devoted to the control of IgG glycosylation has led to the emergence of new concepts and ideas that have been readily translated into the clinic. It has led over the last years to the engineering of a new generation of therapeutic mAbs with enhanced effector functions. Some of these antibodies have already entered clinical trials and one can think that it will be possible to evaluate in a near future whether a significant clinical benefit is obtained without encountering severe side-effects due to the enhanced ability of these mAbs to interact with FcγR. Finally, it is likely that the recent insights gained into the IVIg mechanisms of action will translate also in the generation of new engineered molecules.
